# Supplementary material for: Design, Synthesis, and Biological Evaluation of Novel MAO-A Inhibitors Targeting Lung Cancer
Source: Molecules. 2022 Apr 30;27(9):2887. doi: 10.3390/molecules27092887 (PMC9103226; doi:10.3390/molecules27092887)
Supplement: Supplementary file 1 [file molecules-27-02887-s001.zip › molecules-1673598-supplementary.pdf]

Design, Synthesis, and Biological Evaluation of Novel MAO-A Inhibitors Targeting Lung Cancer

Sanaa Bardaweel [1,*], Reem Aljanabi [1], Dima Sabbah [2] and Kamal Sweidan [3]

1 Department of Pharmaceutical Sciences, School of Pharmacy, The University of Jordan, Amman 11942, Jor-dan; aljanabireem@live.com
2 Department of Pharmacy, Faculty of Pharmacy, Al-Zaytoonah University of Jordan, P.O. Box 130, Amman 11733, Jordan; dima.sabbah@zuj.edu.jo
3 Department of Chemistry, The University of Jordan, Amman 11942, Jordan; k.sweidan@ju.edu.jo
* Correspondence: s.bardaweel@ju.edu.jo; Tel.: +00-(96)-265355000 (ext. 23318)

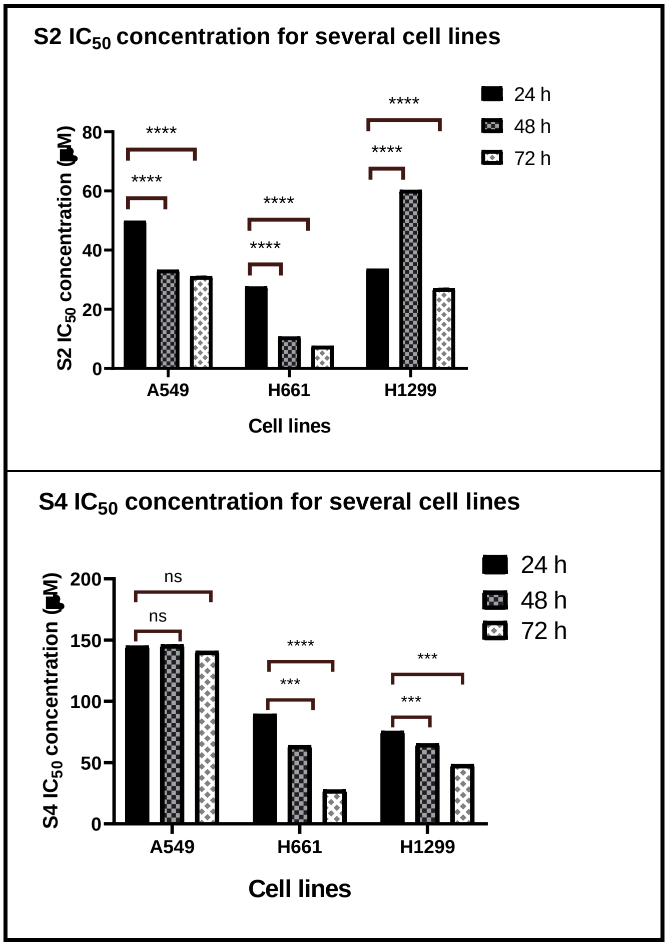

Figure S1. Time-dependent inhibition of A549, H661, and H1299 lung cancer cells at 24, 48, 72 hours treatment duration.

Data shown represent IC_{50}. IC_{50} was calculated using Prism software. Cells were cultured and allowed to attach overnight. Next day, cells were treated with different concentrations of S2, and S4. Afterwards, the cell viability was determined using MTT assay. Each experiment was performed in doublicate and repeated at least four times independently. P-value <0.05 indicates statistical significance in-comparison to IC_{50} at 24h treatment, while asterisk: ns (not-significant) $P > 0.05$; * $P \leq 0.05$; ** $P \leq 0.01$; *** $P \leq 0.001$; **** $P \leq 0.0001$ (according to GraphPad prism 9). μM: micromolar.

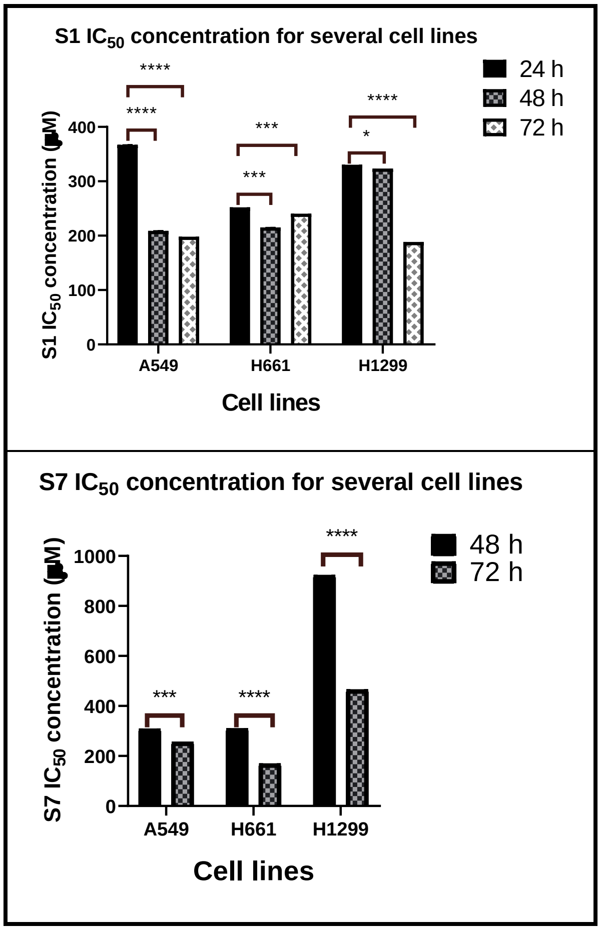

Figure S2. Time-dependent inhibition of A549, H661, and H1299 lung cancer cells at 24, 48, 72 hours treatment duration.

Data shown represent IC_{50}. IC_{50} was calculated using Prism software. Cells were cultured and allowed to attach overnight. Next day, cells were treated with different concentrations of S1, and S7. Afterwards, the cell viability was determined using MTT assay. P-value <0.05 indicates statistical significance in-comparison to IC_{50} at 48h treatment, while asterisk: ns (not-significant) $P > 0.05$; * $P \leq 0.05$; ** $P \leq 0.01$; *** $P \leq 0.001$; **** $P \leq 0.0001$ (according to GraphPad prism 9). µM: micromolar.

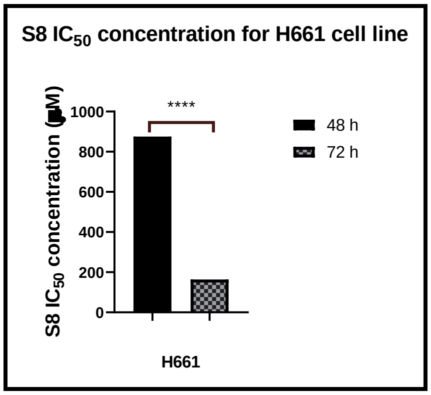

Figure S3. Time-dependent inhibition of H661 lung cancer cells at 48, and 72 hours treatment duration.

Data shown represent IC_{50}. IC_{50} was calculated using Prism software. Cells were cultured and allowed to attach overnight. Next day, cells were treated with different concentrations of S8. Afterwards, the cell viability was determined using MTT assay. P-value <0.05 indicates statistical significance in-comparison to IC_{50} at 48h treatment, while asterisk: ns (not-significant) $P > 0.05$; * $P \leq 0.05$; ** $P \leq 0.01$; *** $P \leq 0.001$; **** $P \leq 0.0001$ (according to GraphPad prism 9). µM: micromolar.

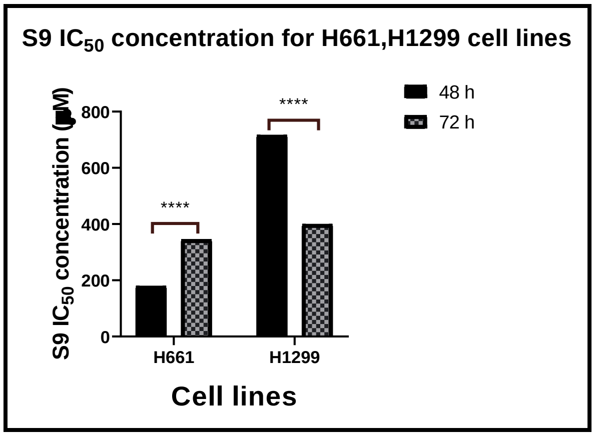

Figure S4. Time-dependent inhibition of H661, and H1299 lung cancer cells at 48, and 72 hours treatment duration.

Data shown represent IC_{50}. IC_{50} was calculated using Prism software. Cells were cultured and allowed to attach overnight. Next day, cells were treated with different concentrations of S9. Afterwards, the cell viability was determined using MTT assay.

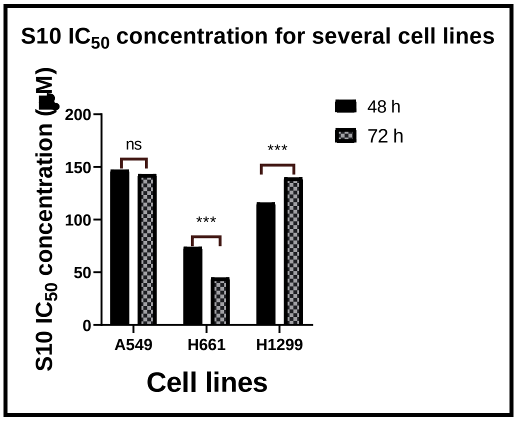

Figure S5. Time-dependent inhibition of A549, H661, and H1299 lung cancer cells at 48, and 72 hours treatment duration.

Data shown represent IC50. IC50 was calculated using Prism software. Cells were cultured and allowed to attach overnight. Next day, cells were treated with different concentrations of S10. Afterwards, the cell viability was determined using MTT assay. P-value <0.05 indicates statistical significance in-comparison to IC50 at 48h treatment, while asterisk: ns (not-significant) P > 0.05; * P ≤ 0.05; ** P ≤ 0.01; *** P ≤ 0.001; **** P ≤ 0.0001 (according to GraphPad prism 9). µM: micromolar.

Table S1. The 50% inhibitory concentration (IC_{50}) values for MAO-A inhibitors in fibroblast cells at 24, 48, 72 h Experiments were carried out in duplicates in 4 independent times (n=8). SD did not exceed 5%. SD: standard deviation, h: hour; µM: micromolar.

Time	Fibroblast cells				
	S2 (IC_{50} µM)	S4 (IC_{50} µM)	S1 (IC_{50} µM)	S7 (IC_{50} µM)	S10 (IC_{50} µM)
**24 h**	>150	>200	>600	>1000	>4000
**48 h**	>150	>200	>600	>1000	>4000
**72 h**	>100	>200	>600	>1000	>4000

^{1}H-NMR, ^{13}C-NMR, DEPT ^{13}C-NMR, and IR spectra of **S7**

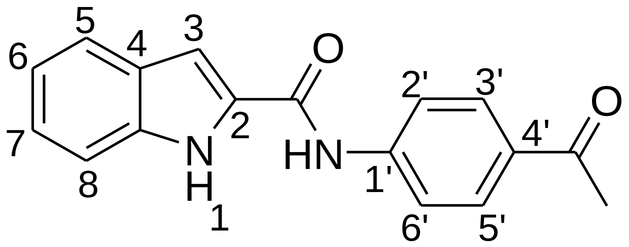

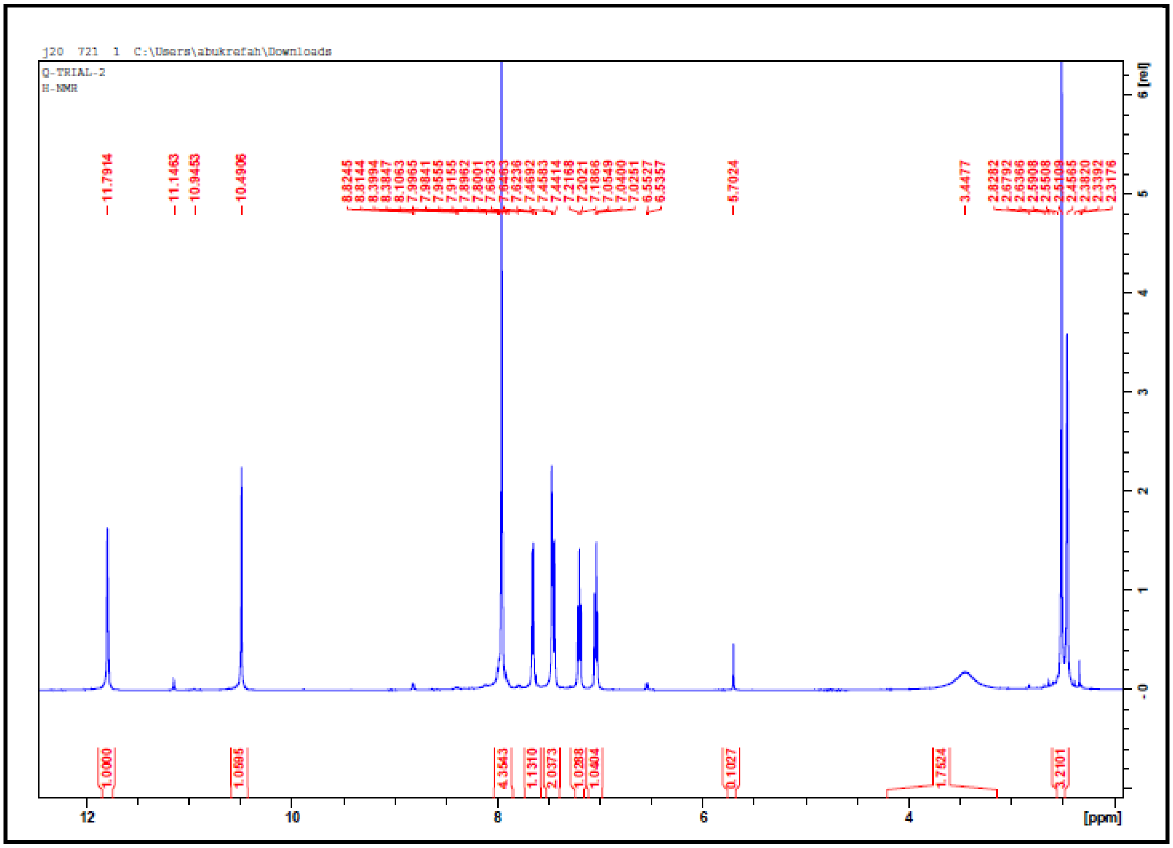

The ^{1}H-NMR spectrum of compound **S7**.

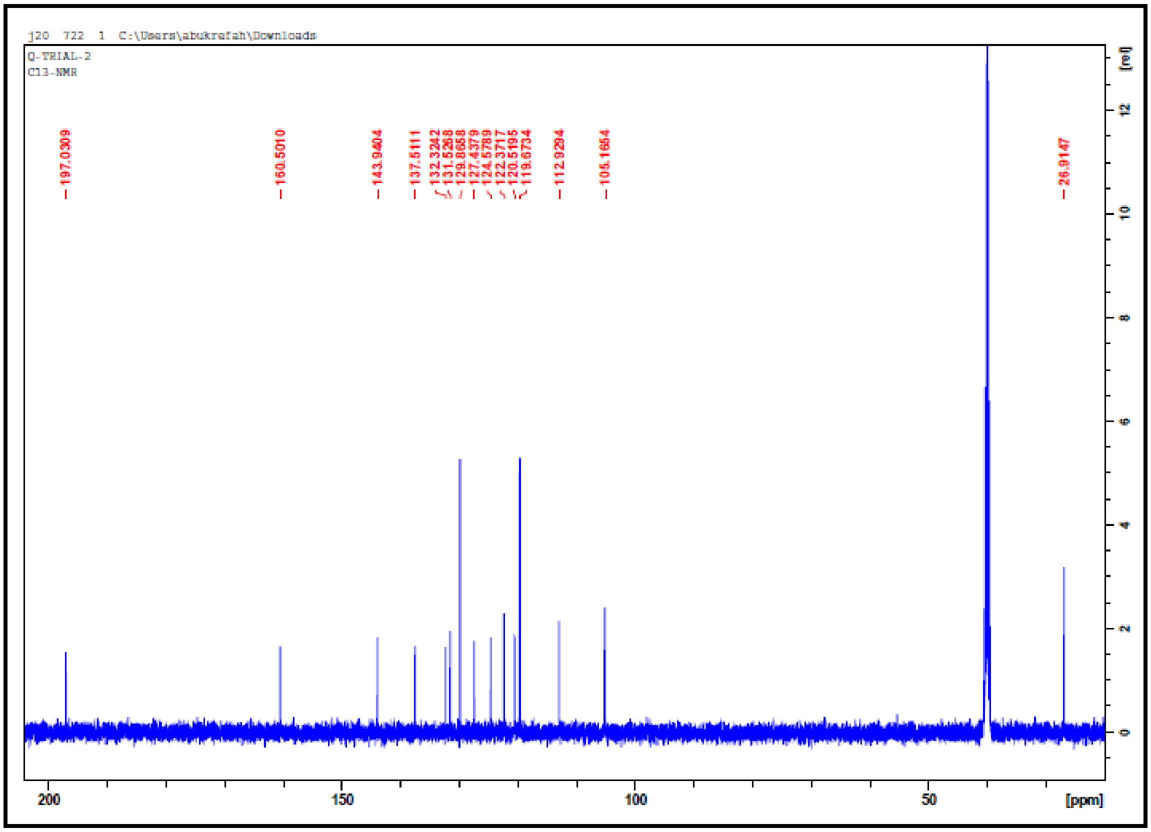

The ^{13}C-NMR spectrum of compound **S7**.

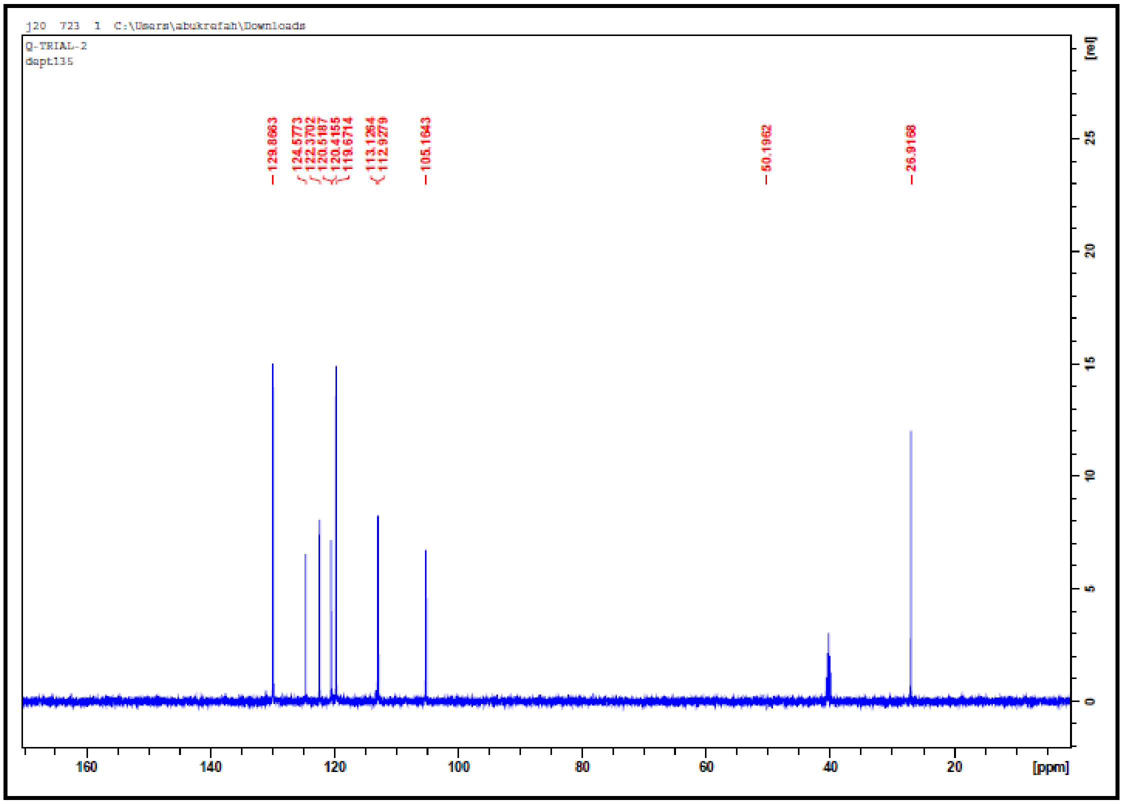

The DEPT ^{13}C-NMR spectrum of compound **S7**.

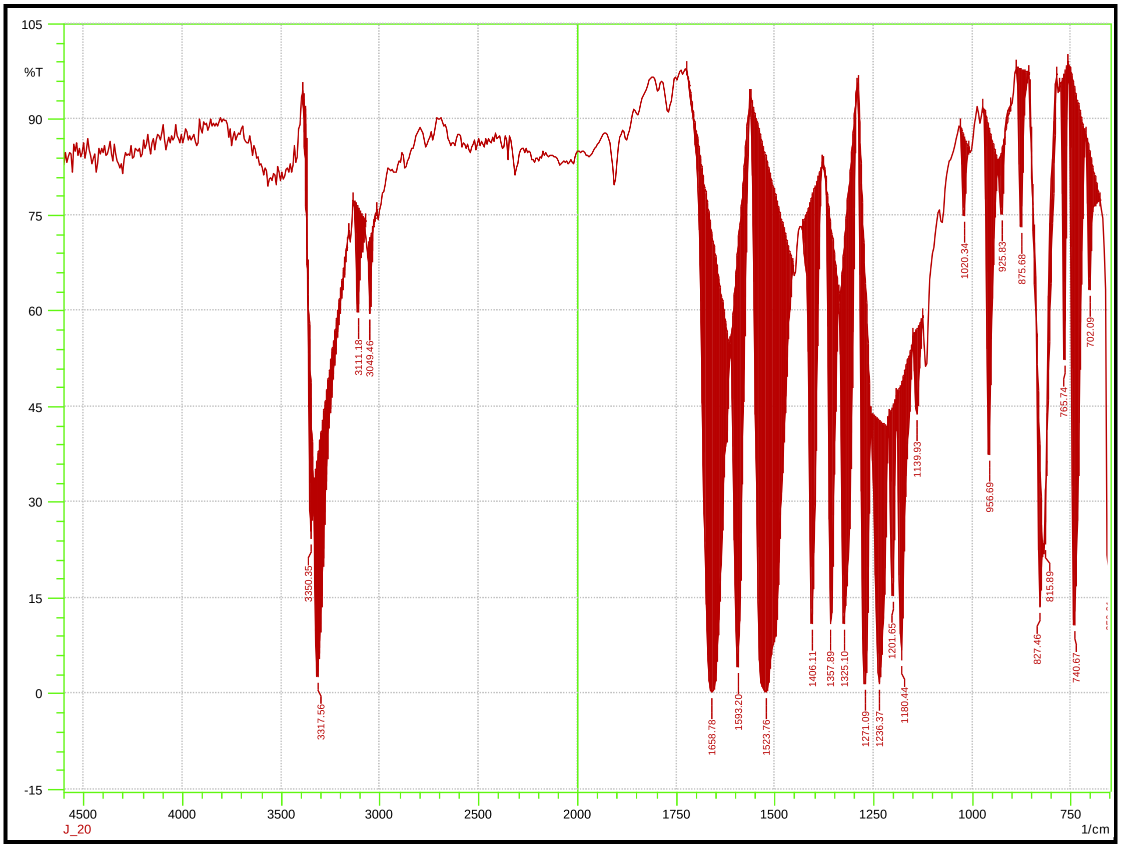

The FT-IR spectrum of **S7**

^{1}H-NMR, ^{13}C-NMR, DEPT ^{13}C-NMR, and IR spectrum of **S8.**

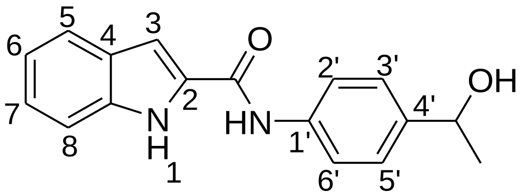

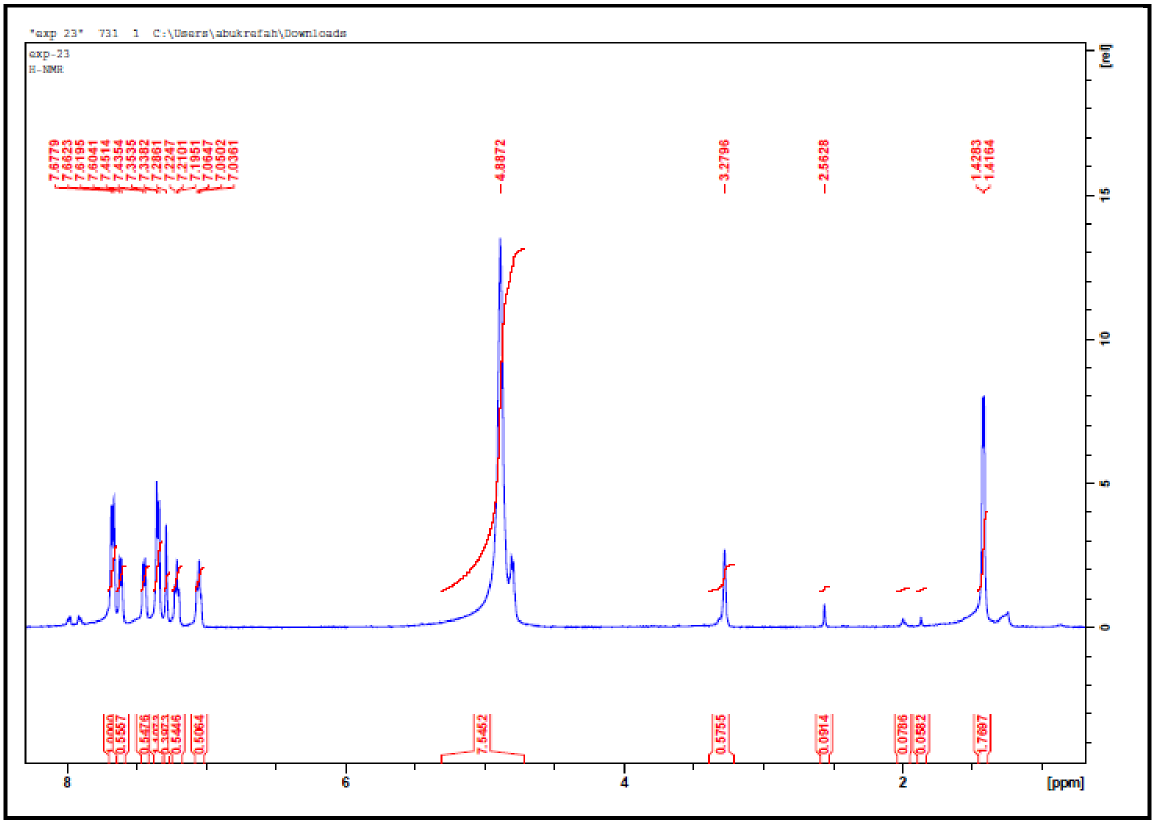

The ^{1}H-NMR spectrum of compound **S8**.

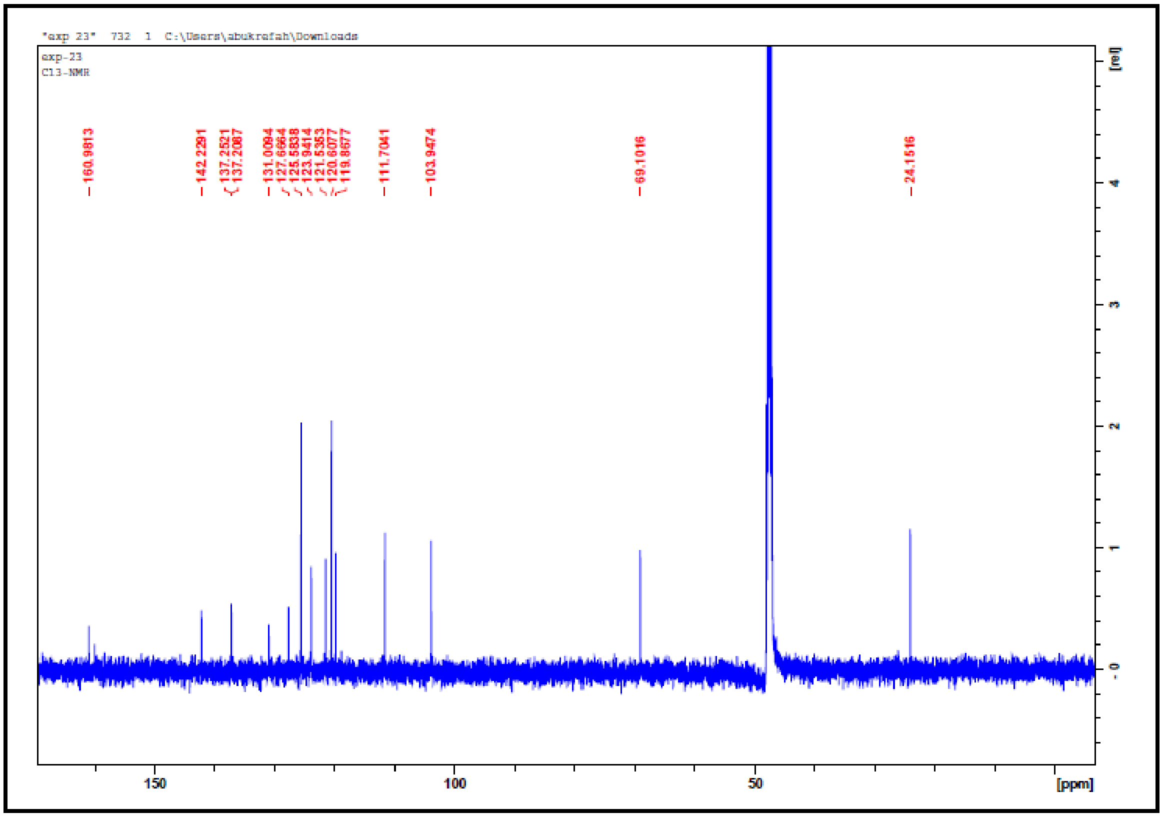

The ^{13}C-NMR spectrum of compound **S8**.

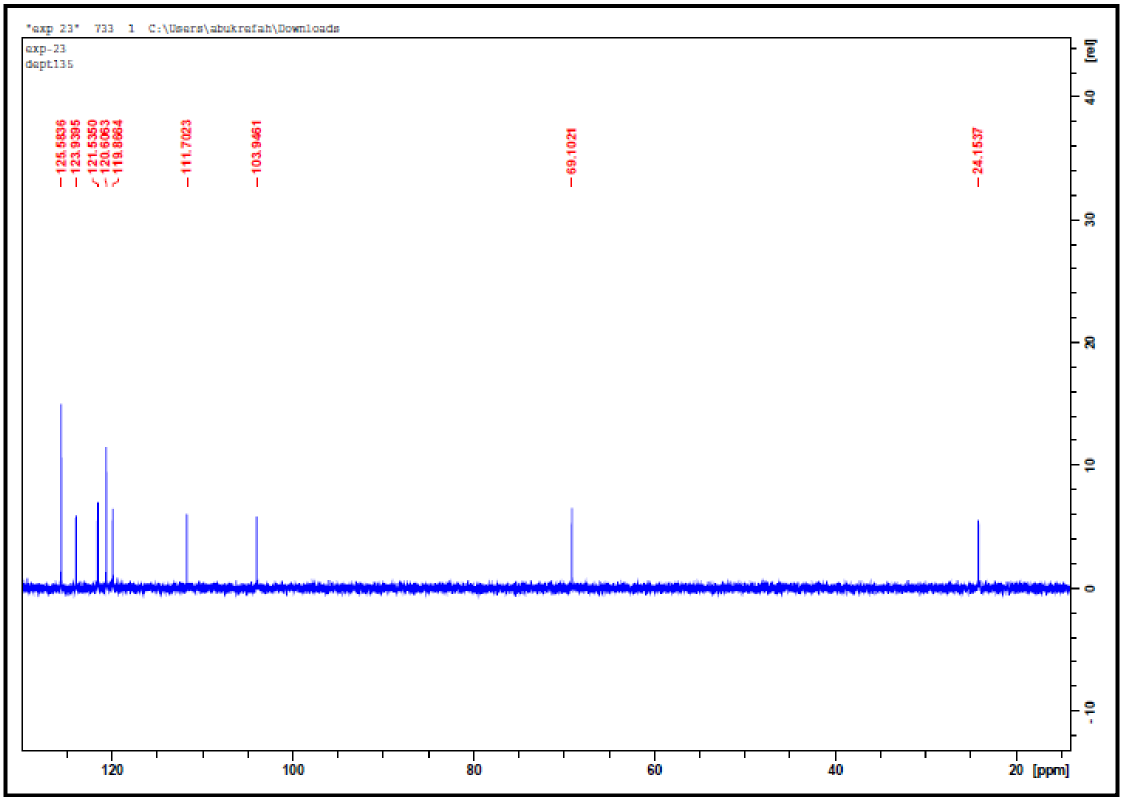

The DEPT ^{13}C-NMR spectrum of compound **S8**.

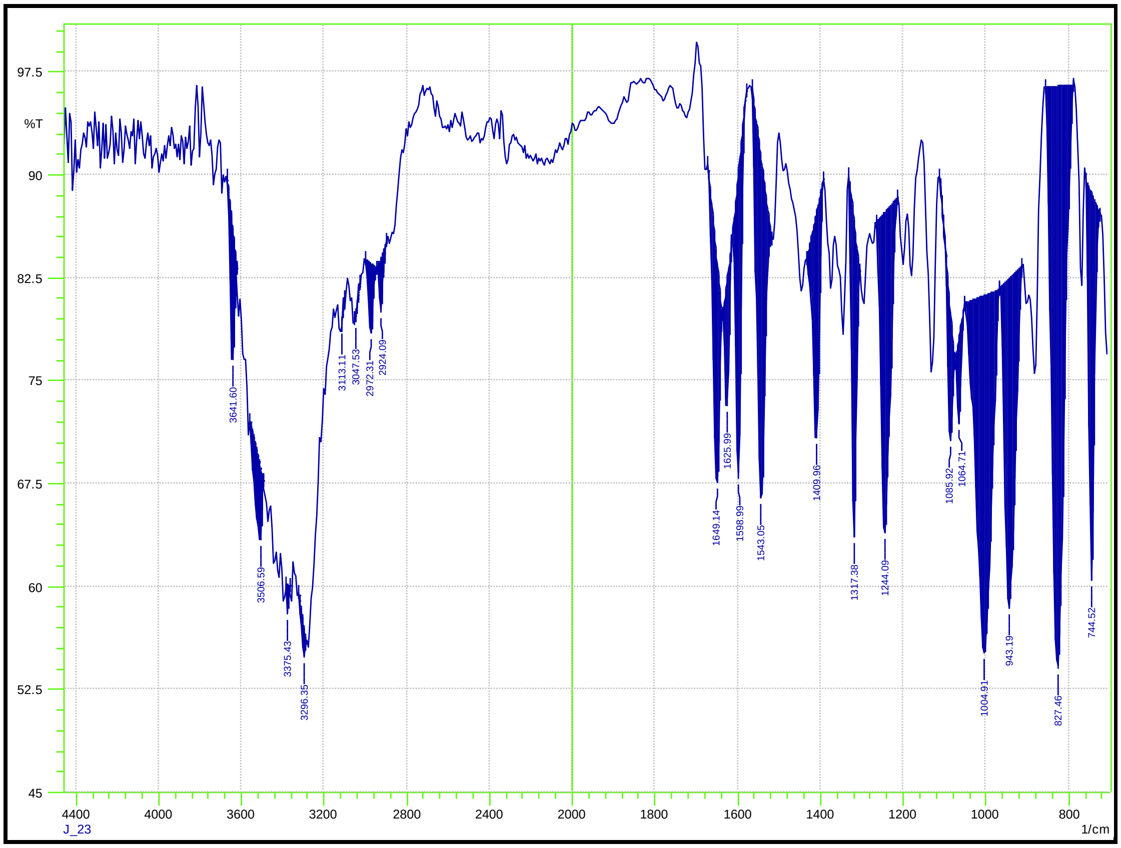

The FT-IR spectrum of **S8**.

^{1}H-NMR, ^{13}C-NMR, DEPT ^{13}C-NMR, and IR spectrum of **S9**

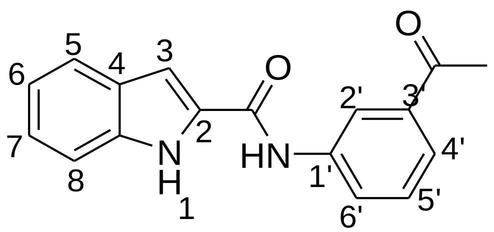

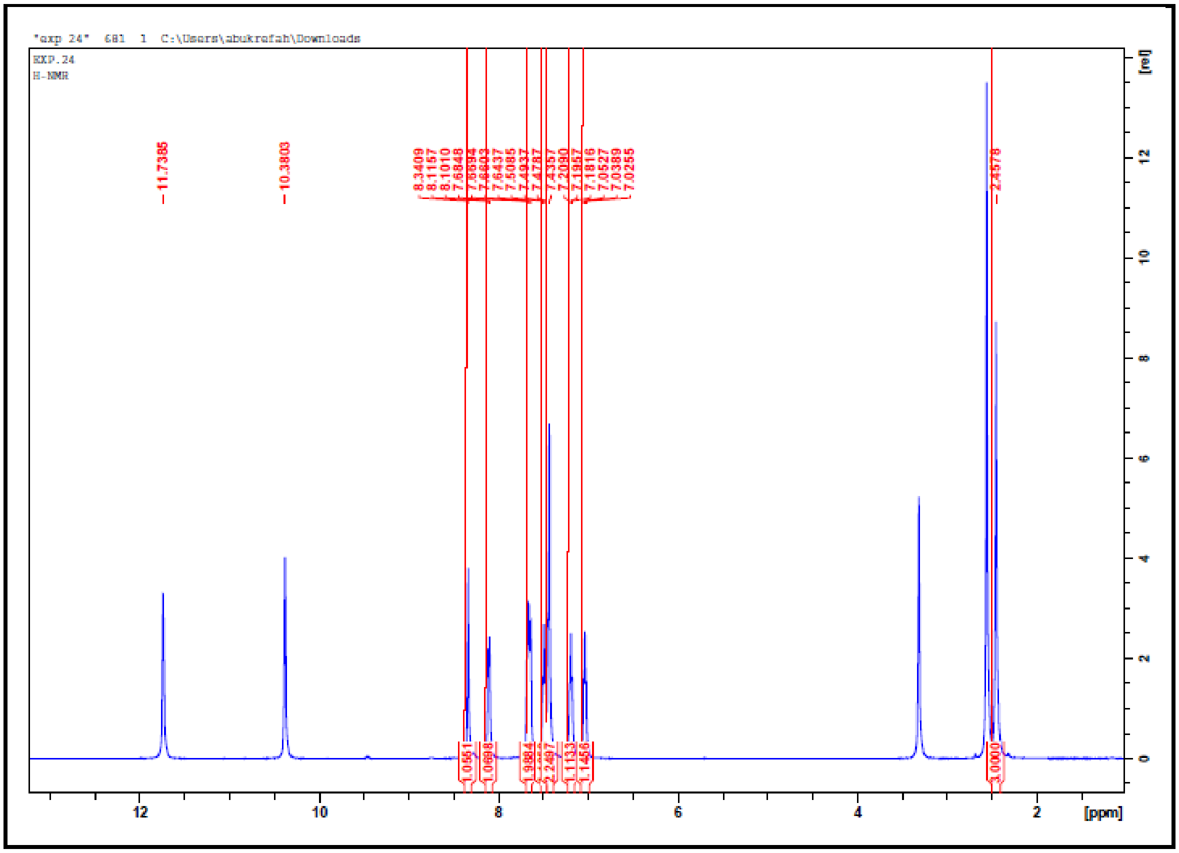

The ^{1}H-NMR spectrum of compound **S9**.

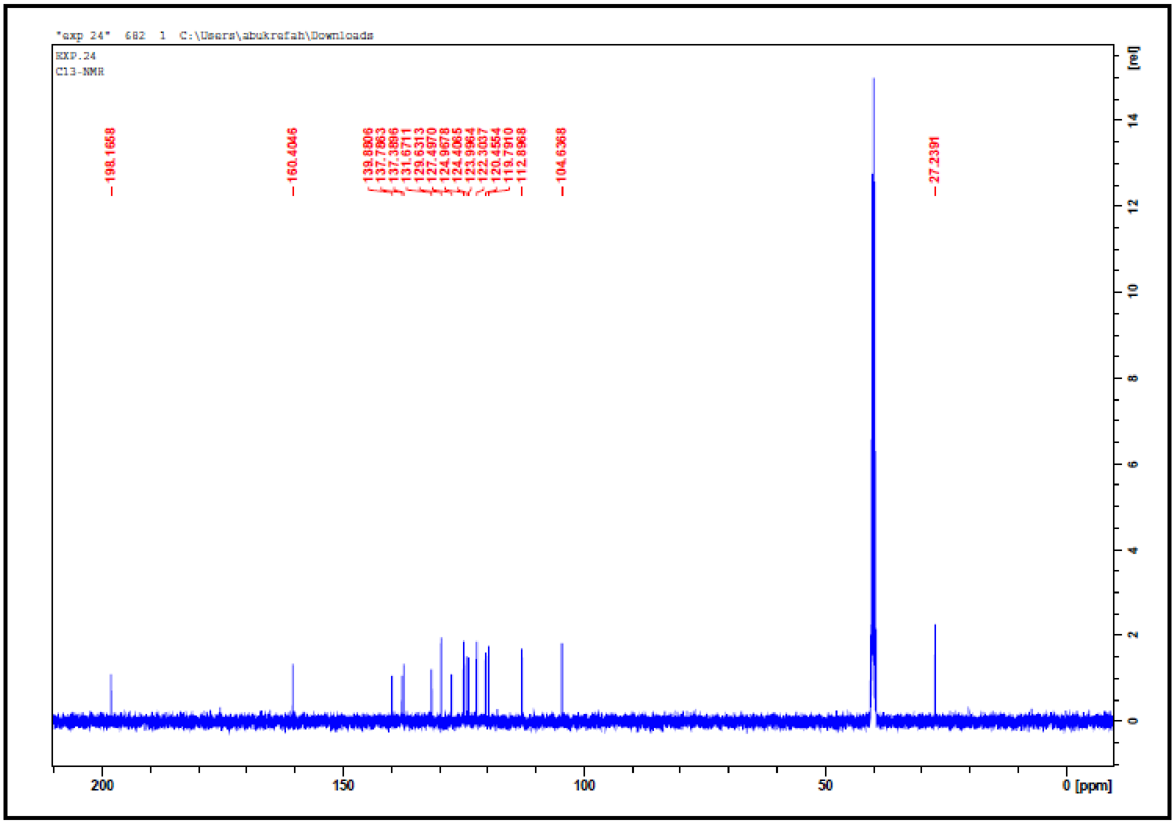

The ^{13}C-NMR spectrum of compound **S9**.

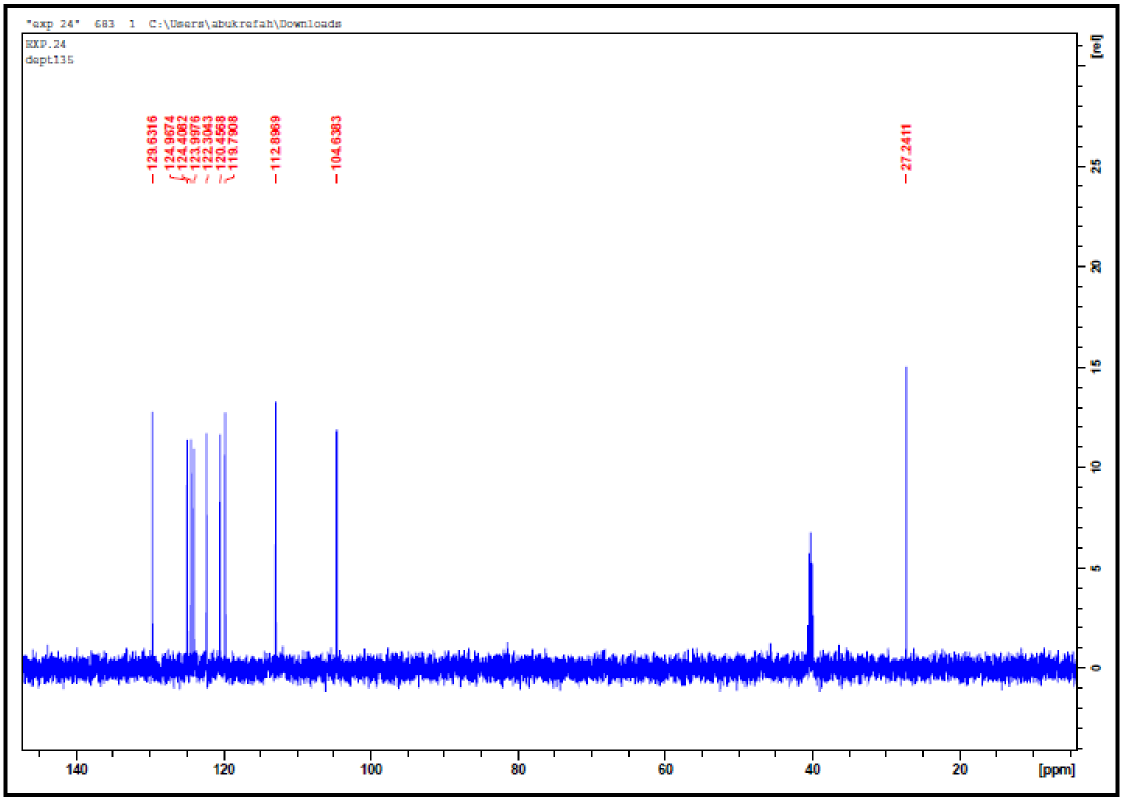

The DEPT ^{13}C-NMR spectrum of compound **S9**.

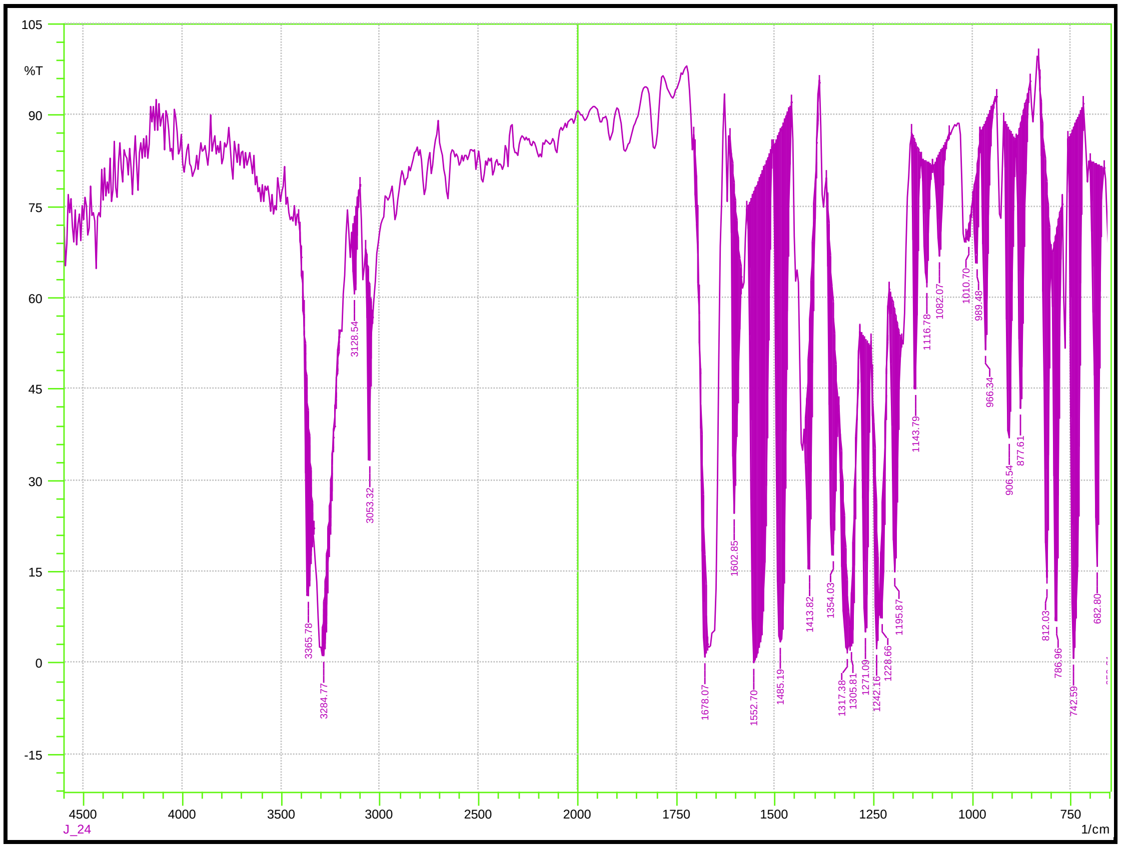

The FT-IR spectrum of **S9**.

^{1}H-NMR, ^{13}C-NMR, DEPT ^{13}C-NMR, and IR spectrum of **S10**.

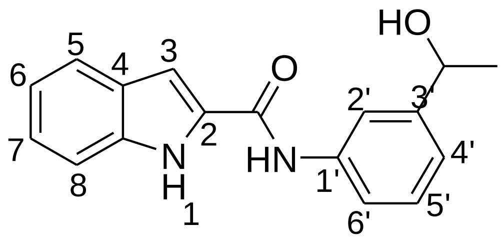

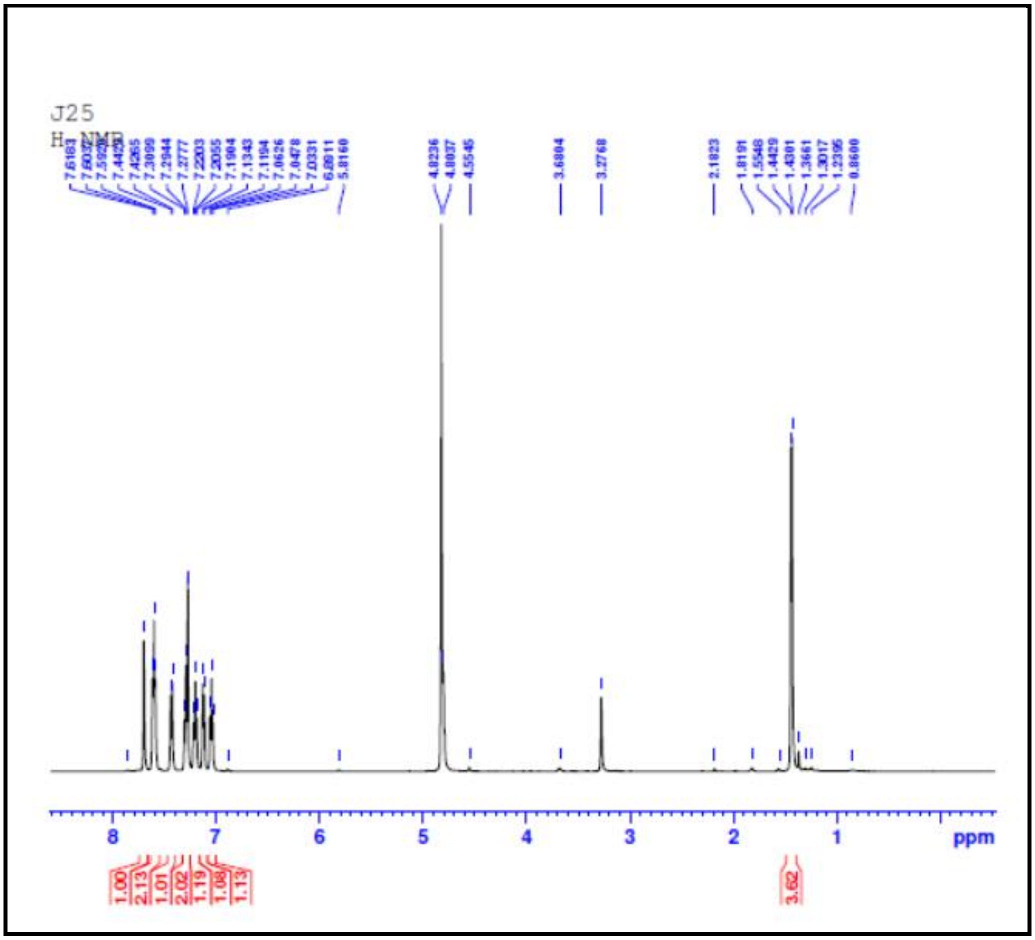

The ^{1}H-NMR spectrum of compound **S10**.

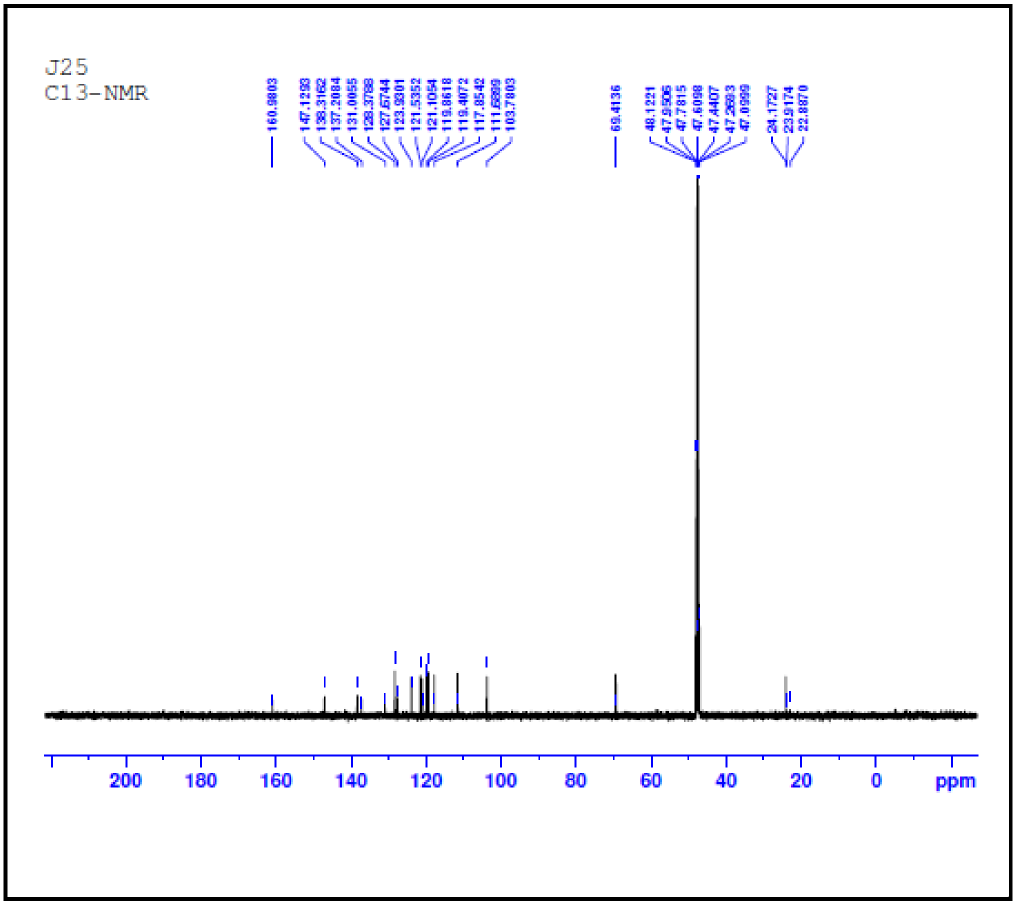

The ^{13}C-NMR spectrum of compound **S10**.

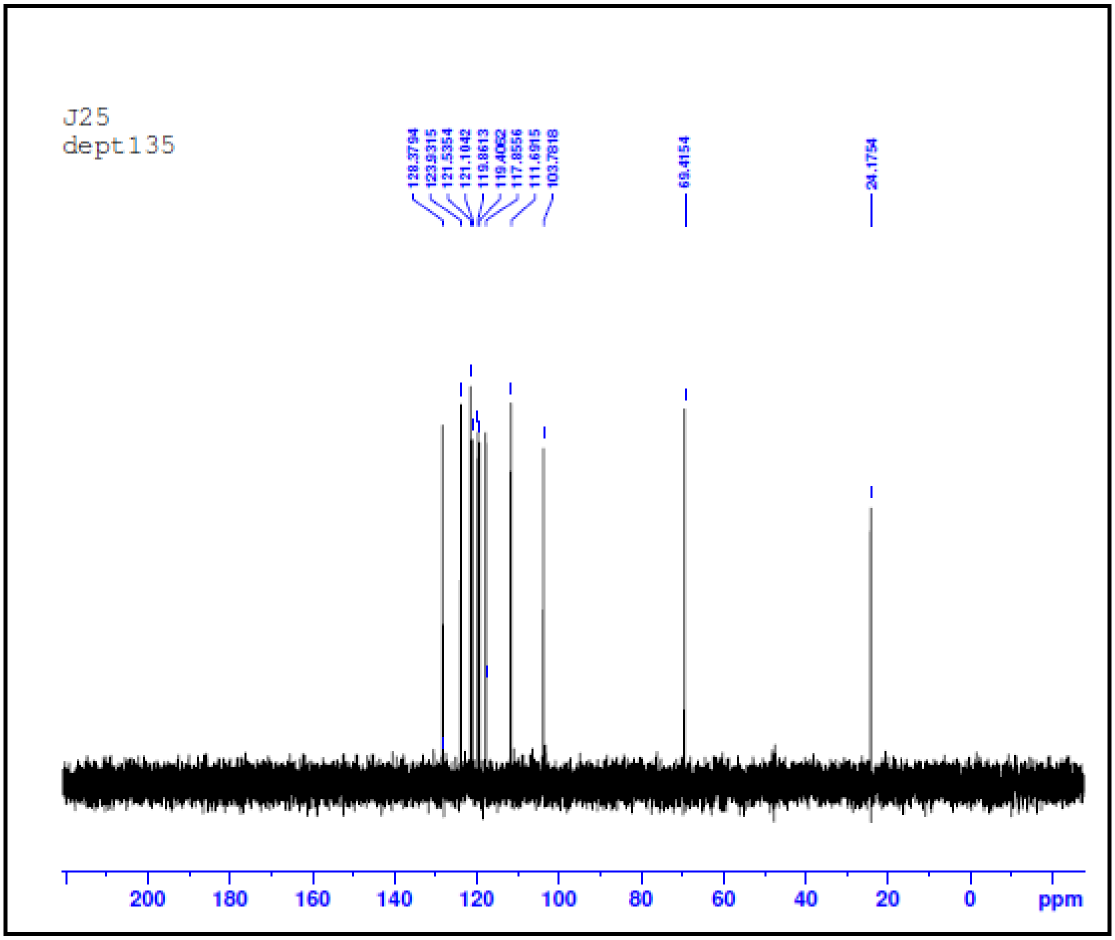

The DEPT ^{13}C-NMR spectrum of compound **S10**.

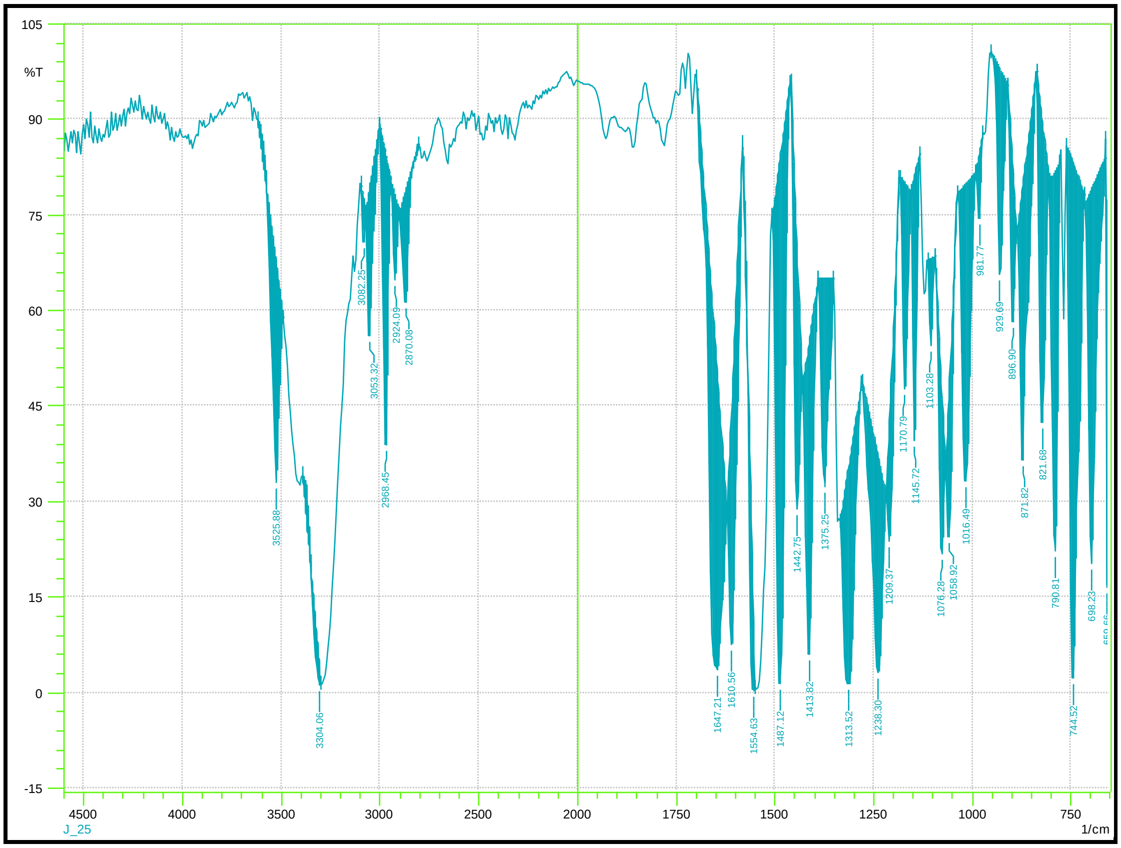

The FT-IR spectrum of **S10**.
